# GenderMedDB: an interactive database of sex and gender-specific medical literature

**DOI:** 10.1186/2042-6410-5-7

**Published:** 2014-05-23

**Authors:** Sabine Oertelt-Prigione, Björn-Oliver Gohlke, Mathias Dunkel, Robert Preissner, Vera Regitz-Zagrosek

**Affiliations:** 1 Institute of Gender in Medicine, Charité-Universitätsmedizin Berlin, Hessische Str. 3/4, Berlin 10117, Germany; 2 German Center for Cardiovascular Research (DZHK), Berlin, Germany; 3 Structural Bioinformatics Group, Charité-Universitätsmedizin Berlin, Berlin 13125, Germany; 4 German Cancer Consortium (DKTK), Heidelberg 69120, Germany; 5 Center for Cardiovascular Research (CCR), Charité-Universitätsmedizin Berlin, Berlin 10115, Germany

**Keywords:** Sex differences, Gender differences, Database, Archive, Search engine, Literature

## Abstract

**Background:**

Searches for sex and gender-specific publications are complicated by the absence of a specific algorithm within search engines and by the lack of adequate archives to collect the retrieved results. We previously addressed this issue by initiating the first systematic archive of medical literature containing sex and/or gender-specific analyses. This initial collection has now been greatly enlarged and re-organized as a free user-friendly database with multiple functions: GenderMedDB (http://gendermeddb.charite.de).

**Description:**

GenderMedDB retrieves the included publications from the PubMed database. Manuscripts containing sex and/or gender-specific analysis are continuously screened and the relevant findings organized systematically into disciplines and diseases. Publications are furthermore classified by research type, subject and participant numbers. More than 11,000 abstracts are currently included in the database, after screening more than 40,000 publications. The main functions of the database include searches by publication data or content analysis based on pre-defined classifications. In addition, registrants are enabled to upload relevant publications, access descriptive publication statistics and interact in an open user forum.

**Conclusions:**

Overall, GenderMedDB offers the advantages of a discipline-specific search engine as well as the functions of a participative tool for the gender medicine community.

## Background

Gender medicine is a newly established, yet rapidly growing subdiscipline of clinical medicine, which investigates the differences between women and men in epidemiology, pathophysiology, clinical features, management and outcomes of disease [1-5]. Aspects of preventive and health behaviour, access to health care, as well as socioeconomic influences are also investigated [6-9].

Previous efforts to develop specific search filters for open search engines have been described [10], yet none of these leads to a permanent collection of literature. Thus, given the difficulties in retrieving literature that investigates sex and gender differences, we have previously launched a pilot project, which led to the development of the first archive of subject-specific literature [11]. The collection featured more than 4,000 publications in nine clinical disciplines and was available in an archive form. This archive has now been transformed in an interactive database, GenderMedDB, which enables the retrieval of pre-selected publications that contain sex and gender-specific analyses. More than 11,000 abstracts, which are the product of a preliminary screening of more than 40,000 publications, are currently available for consultation. No other database of this extent is available in the field of gender medicine, although efforts to compile collections of relevant manuscripts are currently underway in other institutions [12].

In biomedicine, ‘sex’ and ‘gender’ are frequently mixed and used interchangeably, although in theory, two distinct entities are described. Sex is supposed to describe only biological differences, while gender includes social, cultural and economic aspects, as well as the incorporation of power dimensions. The second is frequently neglected in medicine conceptually, yet the term ‘gender’ is used without distinction. In the planning process of the database, we evaluated the option of a strict distinction between sex-specific and gender-specific publications. We came to the conclusion that in medicine, this distinction is frequently impossible, since even genetic and epigenetic analysis [13] must take societal influences into account. Thus, although the terminology is frequently used imprecisely, we refused to perform a forceful segregation of the two, which would lead to the loss of valuable information.

The database has been specifically designed to aid researchers with the retrieval of potentially relevant literature. Searches can focus on publication data or employ pre-selected categories. In addition, researchers have the option to actively input publications that have not been identified through the search tool, as well as the opportunity to access an open forum, which allows for exchange between specialists in the field and interested colleagues.

## Construction and content

### Construction

**Figure 1:**
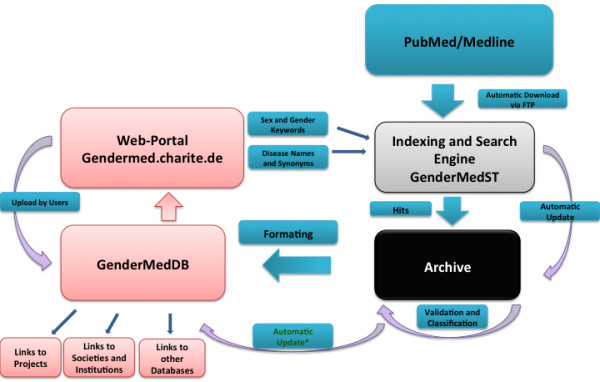
**Workflow in GenderMedDB.** An automatization was implemented to constantly update the data included in the database. In the first step of this pipeline, the complete PubMed database is downloaded via FTP server (ftp://ftp.ncbi.nlm.nih.gov/) of NCBI (National Center for Biotechnology Information) by the LingPipe package. Data are screened, indexed and transferred to the validation table of the database. Pre-selected publications are validated by two researchers and integrated into the GenderMedDB database. Users of GenderMedDB are also enabled to upload publications to the frontend, which will be incorporated into the database after quality control and integration of the categorization.

The GenderMedDB dataset is based on publications from the PubMed database of biomedical literature. The process of download and indexing of articles and the inclusion criteria have been published elsewhere [11]. Briefly, PubMed/Medline data is downloaded from the NCBI FTP site and stored in xml format. Using the software packages Lucene [14] and LingPipe [15], all articles with English abstracts are indexed and publications with potentially relevant content describing sex and/or gender differences are pre-selected. No time limitation has been defined. To define potential relevance, a combination of gender- and disease-related terms is searched in combination. Specialties and diseases have been selected in cooperation with experts in the field based on epidemiological relevance and availability of sex/gender-specific research. Using rule-based methods, like e.g. distance or frequency of keywords, the program generates a score for each hit based on the match rate of the inclusion criteria, position in the text and distance between relevant terms. Lucene uses different types of queries, e.g. term queries, phrase queries, Boolean query and proximity queries for this search approach. Terms are combined, resulting in a number of query outputs for every disease. The indexed data are then dynamically analysed by a search engine, GenderMedST, written in Java (Oracle, Redwood City, CA, USA), which results in a sql file containing the text mining hits. The results are sorted using LingPipe to improve overall classification and to avoid identical coding and spelling. Data are then evaluated by two researchers and uploaded into a relational MySQL (Oracle, Redwood City, CA, USA) database, GenderMedDB (Figure 1).

Web access to the database is enabled via Apache HTTP Server through the website http://gendermeddb.charite.de. For optimal use, recent browser versions of the most commonly used products are recommended. Configuration match was tested for the latest versions of Mozilla Firefox, Google Chrome, Microsoft Internet Explorer and Apple Safari to guarantee reliability in output. Browsers have to be JavaScript enabled.

The database was built and normalized to the third normal form, and large tables were divided into smaller ones to minimize redundancy and dependency (Additional file 1: Figure S1).

### Content

**Figure 2:**
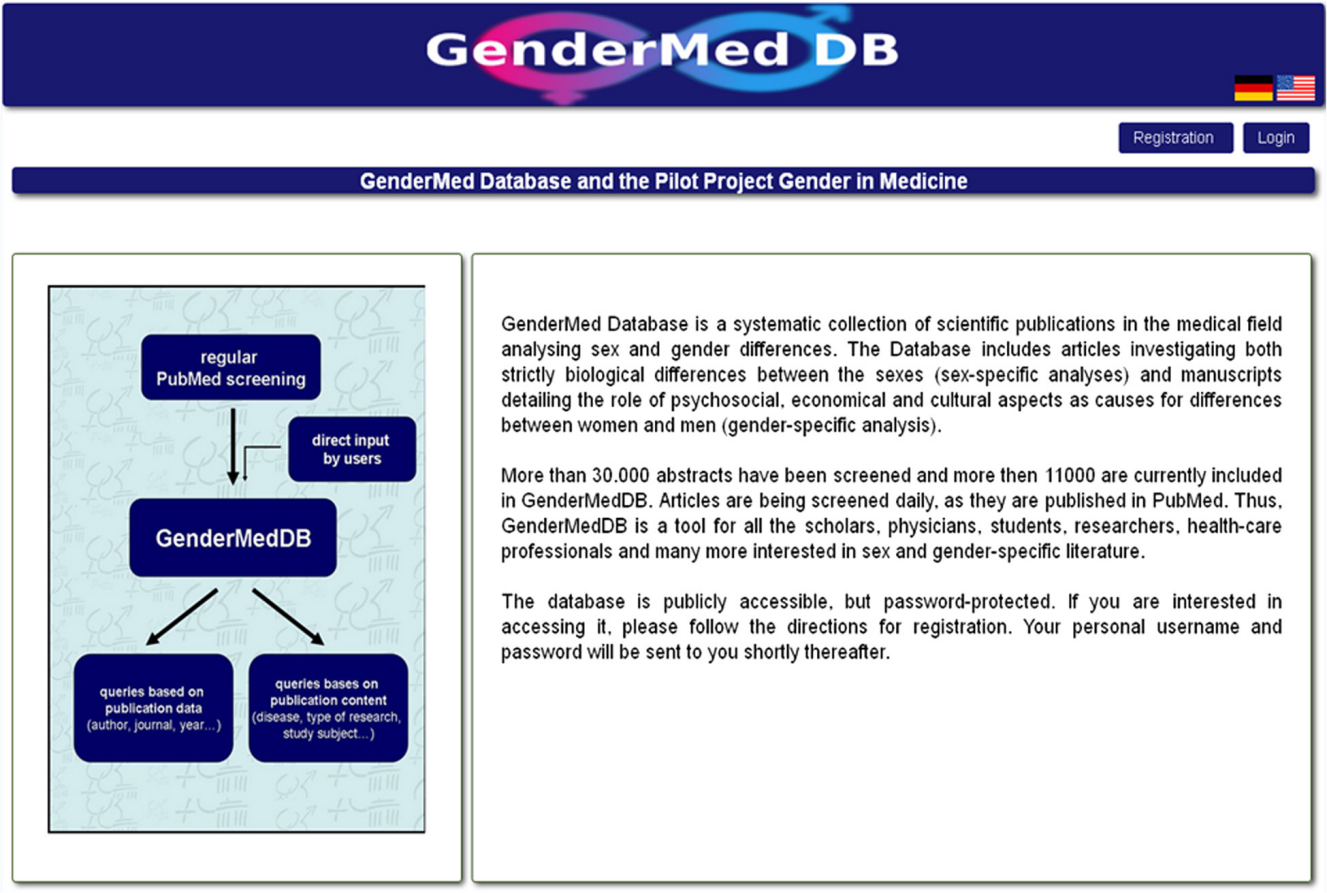
**Screenshot of the frontend of GenderMedDB after login.** All functions are presented in a linear fashion. Dropdown menus lead to the connected subheadings.

Features of the database are presented in a linear fashion (Figure 2). Selection buttons lead to the following options: searches, statistics, forum and upload function, as well as utilities, such as FAQs, links and management windows.

#### Searches

Two options are available for searching: selection by publication data, such as authors, journal and publication year, or selection by content. The latter one gives users the opportunity to employ pre-selected categories, such as discipline, disease, type of research, subject of research and others. A search by specific terms within the abstract is also possible. The output window will present the retrieved results and a link to the publication on the user's institutional PubMed interface, allowing retrieval of full-text articles if institutional access is granted.

#### Statistics

Descriptive statistics detailing publication trends, distribution within disciplines, study subjects and number of study participants are available in each category. This information is structured in three tiers: information about the complete database, information within each discipline and information about selected diseases. For example, after selecting ‘Endocrinology’ in the full database section, it is possible to advance to ‘Diabetes’ and then obtain the following information: since 1976, 894 publications including sex- and gender-specific analyses have been published in PubMed; the majority (>450) included 150 or more participants, and 82% of these publications detail research on human subjects.

The statistics are dynamic and updated in real time whenever new publications are added to the database.

#### Forum

The forum section allows users to post queries, comments and critiques to the database, as well as personal research questions and requests for intra- and transdisciplinary consultation, searches for collaboration partners and information about events, seminars and publications relevant to the field of gender medicine.

#### Upload

The upload function gives users the opportunity to upload manuscripts that have not been included in the database yet. Information can be uploaded stating the PubMedID of the publication or by indicating authors, the publishing journal or the title of the manuscript. This secondary option also allows the user to classify the uploaded manuscript within pre-selected categories (specialty, disease, type of research, etc.).

## Utility and discussion

Users can access the database freely after initial registration (gendermeddb.charite.de). We opted for a mandatory registration to guarantee users some privacy protection in using the forums. The initial user interface offers a visual representation of all the databases main functions (Figure 2), as illustrated in the ‘Content’ section. The interface has been designed in a user-friendly and intuitive fashion and allows direct access to all database options.

In designing the database, the specific needs of the gender medicine community have been used as a blueprint and its structure addresses some of the issues in gender medicine while offering several opportunities for constructive interaction. The primary function of GenderMedDB is its use as a search engine. Selective searches are enabled in two ways: either by using keywords or by applying pre-selected categories. The main advantage of this collection of literature is the significant reduction in the time and effort needed when using a conventional search engine. GenderMedDB retrieves tens to hundreds of articles within selected study areas, offering a meaningful overview about the publications in the field. Using any conventional search engine, one would have to sift through thousands of manuscripts to achieve the same yield. Thus, although the database cannot identify all possible articles related to the query, it will present only publications including sex- or gender-specific research allowing for a significant overview and directing further searches.

In addition to the search function, GenderMedDB provides descriptive statistics about all the included publications, from general to disease-specific representations. This function displays publication trends over the last three decades and allows the identification of neglected areas along with potential areas of expansion. These outputs represent the ideal tool for inter-disciplinary analyses, as well as a background for research proposals and grant reviewing as they offer an overview of a single field or disease in a broader context.

Given that no research algorithm is perfect and no gold standard for the retrieval of sex- and gender-specific publications has been established yet, GenderMedDB also offers its users the opportunity to upload relevant references. The researcher's own findings and noteworthy publications of colleagues can be uploaded and classified, if desired. We envision that this function might achieve different results; it will significantly enrich the database through external support and allow for a more rapid and comprehensive expansion. In addition, it will encourage a participative approach, fostering the vision of GenderMedDB as a product that can, and should, be actively modified and improved by its community of users.

The forum function further enhances the opportunity for active participation by the users. Registered participants can utilize the provided forum to address not only specific issues concerning the database, but also research issues. Questions about research methodology can be shared, collaborations initiated and relevant events publicized.

Although GenderMedDB offers a significant improvement over direct search strategies and a tool for the participation of the users, some aspects have to be taken into consideration. The search algorithm and tool will not be able to identify all possible publications in any field; thus, some articles will not be included although they contain a significant sex- or gender-specific analysis. Nonetheless, we hope that an active use of the upload function by registrants will partially compensate for this in the long run. In addition, we rejected the idea to rate research based on its scientific quality or publication metrics by using these criteria for inclusion/exclusion into the database. The vision behind GenderMedDB is to offer researchers a selected overview of subject-specific publications, but to leave the judgement about their relevance and value to every single user.

## Conclusions

GenderMedDB offers both the functions of a search engine and a participative structure to include all interested stakeholders in the field of gender medicine. Given the extensive and continuously increasing collection of relevant materials and its open structure, large audiences and active use can be expected. Overall, we foresee that GenderMedDB will significantly support the development of a common knowledge base in the field of gender medicine.

## Availability and requirements

The database can be accessed using the following URL: gendermeddb.charite.de. Registration is required prior to first use, yet this is free of any cost to any user.

## Competing interests

The authors declare that they have no competing interests.

## Authors’ contributions

SOP designed the database and website, collected and analysed the data and wrote the manuscript. BOG developed the database, designed the website and wrote the manuscript. MD developed the database and supervised data processing. RP designed the database and reviewed the manuscript. VRZ supervised the database development, reviewed the manuscript and acquired funding for the project. All authors read and approved the final manuscript.

## Supplementary Material

Additional file 1: Figure S1 Schematic representation of the database. The main table of the database (publication information) contains all information related to the publication (authors, title, abstract, journal, etc.). Information about the disease and category is outsourced to separate tables. Newly uploaded publications are stored in a separate table until a specialist marks these publications as relevant. The same workflow is applied to publications which were identified by the text mining approach. Click here for file

## References

[B1] Canto JG Rogers WJ Goldberg RJ Peterson ED Wenger NK Vaccarino V Kiefe CI Frederick PD Sopko G Zheng ZJ NRMI Investigators Association of age and sex with myocardial infarction symptom presentation and in-hospital mortality JAMA 2012 5 8 813 822 22357832 10.1001/jama.2012.199 PMC4494682

[B2] Legato MJ Beyond women's health the new discipline of gender-specific medicine Med Clin North Am 2003 5 5 917 937 vii 10.1016/S0025-7125(03)00063-4 14621324

[B3] Regitz-Zagrosek V Therapeutic implications of the gender-specific aspects of cardiovascular disease Nat Rev Drug Discov 2006 5 5 425 438 10.1038/nrd2032 16672926

[B4] Arain FA Kuniyoshi FH Abdalrhim AD Miller VM Sex/gender medicine: the biological basis for personalized care in cardiovascular medicine Circ J 2009 5 10 1774 1782 10.1253/circj.CJ-09-0588 19729858 PMC2941262

[B5] Huxley RR Woodward M Cigarette smoking as a risk factor for coronary heart disease in women compared with men: a systematic review and meta-analysis of prospective cohort studies Lancet 2011 5 9799 1297 1305 10.1016/S0140-6736(11)60781-2 21839503

[B6] Bird CE Rieker PP Gender matters: an integrated model for understanding men's and women's health Soc Sci Med 1999 5 6 745 755 10.1016/S0277-9536(98)00402-X 10190637

[B7] Dallongevillle J De Bacquer D Heidrich J De Backer G Prugger C Kotseva K Montaye M Amouyel P EUROASPIRE Study Group Gender differences in the implementation of cardiovascular prevention measures after an acute coronary event Heart 2010 5 21 1744 1749 10.1136/hrt.2010.196170 20956490

[B8] Ranji UR Wyn R Salganicoff A Yu H Role of health insurance coverage in women's access to prescription medicines Womens Health Issues 2007 5 6 360 366 10.1016/j.whi.2007.08.004 18042485

[B9] Viera AJ Thorpe JM Garrett JM Effects of sex, age, and visits on receipt of preventive healthcare services: a secondary analysis of national data BMC Health Serv Res 2006 5 15 10.1186/1472-6963-6-15 16504097 PMC1402283

[B10] Moerman CJ Deurenberg R Haafkens JA Locating sex-specific evidence on clinical questions in MEDLINE: a search filter for use on OvidSP BMC Med Res Methodol 2009 5 25 10.1186/1471-2288-9-25 19366443 PMC2678151

[B11] Oertelt-Prigione S Parol R Krohn S Preissner R Regitz-Zagrosek V Analysis of sex and gender-specific research reveals a common increase in publications and marked differences between disciplines BMC Med 2010 5 70 10.1186/1741-7015-8-70 21067576 PMC2993643

[B12] McGregor AJ Templeton K Kleinman MR Jenkins MR Advancing sex and gender competency in medicine: sex & gender women's health collaborative Biol Sex Differ 2013 5 1 11 10.1186/2042-6410-4-11 23724943 PMC3674893

[B13] Regitz-Zagrosek V Sex and gender differences in health. Science & society series on sex and science EMBO Rep 2012 5 7 596 603 10.1038/embor.2012.87 22699937 PMC3388783

[B14] McCandless MH Gospodnetic O Lucene in Action 2008 2 Greenwich: Manning Publications

[B15] Alias-i. LingPipe 4.1.0 2008 [http://alias-i.com/lingpipe]

