# Supplementary figures and images for: GenderMedDB: an interactive database of sex and gender-specific medical literature
Source: Biol Sex Differ. 2014 May 23;5:7. doi: 10.1186/2042-6410-5-7 (PMC4047004; doi:10.1186/2042-6410-5-7)

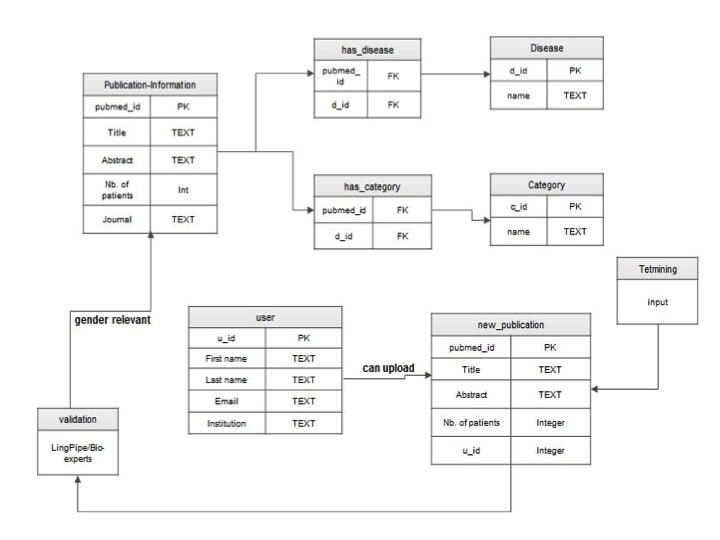

Supplement: Additional file 1: Figure S1 — Schematic representation of the database. The main table of the database (publication information) contains all information related to the publication (authors, title, abstract, journal, etc.). Information about the disease and category is outsourced to separate tables. Newly uploaded publications are stored in a separate table until a specialist marks these publications as relevant. The same workflow is applied to publications which were identified by the text mining approach. [file 2042-6410-5-7-S1.tiff]
